# Retinal arteriolar geometry is associated with cerebral white matter hyperintensities on magnetic resonance imaging

**DOI:** 10.1111/j.1747-4949.2010.00483.x

**Published:** 2010-11-03

**Authors:** Fergus N Doubal, Rosemarie de Haan, Thomas J MacGillivray, Petra E Cohn-Hokke, Bal Dhillon, Martin S Dennis, Joanna M Wardlaw

**Affiliations:** 1 Division of Clinical Neurosciences, University of EdinburghEdinburgh, UK; 2 Academic Medical Centre, University of AmsterdamAmsterdam, The Netherlands; 3 Wellcome Trust Clinical Research Facility, University of EdinburghEdinburgh, UK; 4 Scottish Imaging Network, A Platform for Scientific Excellence (SINAPSE) Collaboration, University of EdinburghEdinburgh, UK; 5 Princess Alexandra Eye Pavilion, University of EdinburghEdinburgh, UK

**Keywords:** cerebral infarction, factors, ischaemic stroke, leukoaraiosis, MRI, risk, stroke

## Abstract

**Background:**

Cerebral small vessel disease (lacunar stroke and cerebral white matter hyperintensities) is caused by vessel abnormalities of unknown aetiology. Retinal vessels show developmental and pathophysiological similarities to cerebral small vessels and microvessel geometry may influence vascular efficiency.

**Hypothesis:**

Retinal arteriolar branching angles or coefficients (the ratio of the sum of the cross-sectional areas of the two daughter vessels to the cross-sectional area of the parent vessel at an arteriolar bifurcation) may be associated with cerebral small vessel disease.

**Methods:**

We performed a cross-sectional observational study in a UK tertiary referral hospital. An experienced stroke physician recruited consecutive patients presenting with lacunar ischaemic stroke with a control group consisting of patients with minor cortical ischaemic stroke. We performed brain magnetic resonance imaging to assess the recent infarct and periventricular and deep white matter hyperintensities. We subtyped stroke with clinical and radiological findings. We took digital retinal photographs to assess retinal arteriolar branching coefficients and branching angles using a semi-automated technique.

**Results:**

Two hundred and five patients were recruited (104 lacunar stroke, 101 cortical stroke), mean age 68-years (standard deviation 12). With multivariate analysis, increased branching coefficient was associated with periventricular white matter hyperintensities (*P*=0·006) and ischaemic heart disease (*P*<0·001), and decreased branching coefficient with deep white matter hyperintensities (*P*=0·003), but not with lacunar stroke subtype (*P*=0·96). We found no associations with retinal branching angles.

**Conclusions:**

Retinal arteriolar geometry differs between cerebral small vessel phenotypes. Further research is needed to ascertain the clinical significance of these findings.

## Introduction

Lacunar or small subcortical ischaemic strokes make up 25% of ischaemic strokes (1) and arise from the occlusion of a single small perforating artery, although the exact aetiology remains uncertain (2). Lacunar strokes are associated with white matter hyperintensities (WMH) (3), which are associated with ageing (4), cognitive impairment and dementia (5); however, the exact aetiology of these WMH is unknown.

The retinal arterioles are of similar size and physiology to the cerebral arterioles (6). Cerebral arteriole sizes are below that which can be visualised reliably using current human imaging techniques but the retina can be photographed directly. Retinal vascular abnormalities are associated with both stroke and white matter disease presence and progression (7–10) and retinal venular (11, 12) and arteriolar (12) widths differ between stroke subtypes. Retinal vessel abnormalities may act as markers for cerebral small vessel disease, although retinal vascular geometry has not been studied in ischaemic stroke subtypes.

The geometry of arterioles may affect the efficiency of circulation (13), that is, the ability of the arteriolar tree to deliver blood to tissue with a minimum total blood volume and with minimal loss of energy at each bifurcation. The branching coefficient of an arteriolar bifurcation measures the change in the total cross-sectional area across the bifurcation. An increased branching coefficient represents wider daughter vessels and a decreased branching coefficient indicates narrower daughters compared with the parent vessel; each may affect the energy required to deliver blood around the body and hence the efficiency of the circulatory system. Some studies suggest that this theory may be biologically true, for example abnormalities in branching coefficient have been associated with cognitive impairment (14), peripheral vascular disease (15) and ischaemic heart disease (IHD) (16).

Retinal arteriolar branching angles represent the angle subtended by the two daughter vessels. A change in the absolute angles or a deviation away from a theoretical optimum branching angle may also affect the circulatory efficiency and studies have also shown associations between branching angles and hypertension (17) and cognitive function (14).

## Hypothesis

If cerebral small vessel disease were due to an intrinsic small vessel abnormality, then patients with cerebral small vessel disease (either lacunar stroke or WMH) would have altered retinal arteriolar branching coefficients and branching angles.

## Methods

### Patients

We prospectively recruited consecutive patients with a clinical syndrome of acute lacunar or mild cortical stroke presenting to our university hospital stroke service between April 2005 and December 2007. We excluded patients with contraindications to MR, haemorrhage, severe stroke and nonstroke diagnoses. An experienced stroke physician examined the patients, assessed stroke severity using the National Institute for Health Stroke Scale (18) and classified patients into lacunar or cortical stroke clinical syndromes according to the Oxfordshire Community Stroke Project classification (19). Patients underwent investigations for stroke as indicated including magnetic resonance imaging (MRI) at presentation. We recorded history of diabetes, hypertension, IHD and peripheral vascular disease. We defined symptomatic carotid stenosis as a relevant stenosis >50% measured with the North American Symptomatic Carotid Endarterectomy Trial (20). We defined atrial fibrillation as either a history of paroxysmal or continuous atrial fibrillation or atrial fibrillation on electrocardiogram.

### Brain imaging

Patients had diagnostic brain MRI at presentation, on a 1·5 T MR scanner (Signa LX; General Electric, GE Company, Fairfield, CT, US) with 22 mT/m maximum strength gradients. Diagnostic MRI included axial diffusion-weighted, T2-weighted, fluid-attenuated inversion recovery (FLAIR) and gradient echo sequences.

### MRI analysis

Magnetic resonance imaging scans were coded for the presence, location and size of the recent infarct and any old infarcts or haemorrhages by a neuroradiologist. A recent infarct was defined as a hyper-intense area on diffusion imaging (with a corresponding reduced signal on apparent diffusion coefficient image processing), with or without an increased signal on FLAIR or T2-weighted imaging, in a distribution compatible with an arterial territory. Lacunar infarcts were in the cerebral hemispheric white matter, basal ganglia or brain stem and <2 cm diameter if recent (subcortical lesions >2 cm were classed as striatocapsular or cortical as they have large artery disease causes). Magnetic resonance imaging scans were coded for deep (lesions not contiguous with the ventricles) and periventricular (lesions contiguous with the ventricles) WMH using the Fazekas scale, which rates lesions in both regions from 0 to 3 (21).

### Stroke subtyping

We defined mild cortical stroke syndrome as a maximum clinical deficit of either: weakness or sensory loss in the face, arm or leg; loss of higher cerebral dysfunction (e.g. dysphasia or neglect); and weakness or sensory loss in the presence of loss of higher cerebral function or homonymous hemianopia suggestive of occipital cortical infarct (19). We defined lacunar stroke syndrome as one of the classical lacunar syndromes (19). We also classified stroke subtype using radiological criteria (i.e. whether the recent infarct on MRI was cortical or lacunar) and used both the clinical and the radiological classification to assign a final stroke subtype classification (19). Where the clinical differed from the radiological classification, the radiological classification took precedence as clinical diagnosis can misclassify up to 20% (22). If no definite recent lesion was visible on the scan, the clinical classification was used. We recorded old lesions but subtyped based on the acute lesion.

### Retinal assessment

Patients had six-field retinal photography (centred on the disc, macula, lateral macula, nasal to the disc, upper arcade and lower arcade) of both eyes, with 1% tropicamide eye, using a Canon CR-DGi digital retinal camera (Canon USA Inc., Lake Success, NY, USA). We selected photographs centred on the optic disc for each eye. Images were analysed within custom-written Matlab software (The Mathworks Inc., Natick, NA, USA) blinded to all clinical and imaging details.

### Branching coefficient assessment

**Fig 1:**
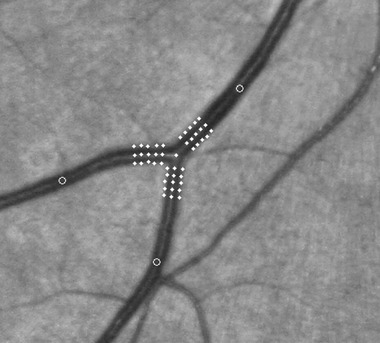
Illustration of vessel tracking across a bifurcation to measure widths for branching coefficient calculation. Please note that for illustrative purposes the vessel demonstrated in the image is a venule. We performed measurements on retinal arterioles.

A trained grader identified the five most proximal measurable arteriolar junctions to the optic disc and used semi-automated computer software to measure the branching coefficient of each bifurcation. The software tracked down each vessel from the centre point of each bifurcation fitting a profile of signal intensity at right angles to the longitudinal axis of the vessel with a Gaussian curve to determine the width of each vessel (Fig. 1). Each profile was manually inspected and rejected if the Gaussian line did not fit well (*r* correlation <0·7). We validated this process with Bland–Altman plots comparing software performance to best human measurement (with a caliper on enlarged images) and found no systematic bias and a mean difference for 50 randomly chosen vessels of 0·006 pixels [95% confidence interval (CI) −3·3 to 3·3 pixels]. We calculated the branching coefficient using the following formula, where *D*_0_ is the parent diameter and *D*_1_ and *D*_2_ the daughter diameters (14):


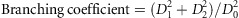


We aimed to measure the branching coefficients of the five most proximal measurable bifurcations to the optic disc but included patients in whom three or more were measurable in our primary analysis. In a prespecified sensitivity analysis, we also analysed patients who had at least five branching coefficient measurements. We avoided assuming that branching coefficients within each eye were normally distributed by taking the median branching coefficient for each eye. There was good within-patient correlation between left and right eyes (Pearson's correlation coefficient 0·53 for 32 randomly chosen patients) and we randomly chose an eye to measure. The intrarater intraclass correlation coefficient for a random sample of 10 images assessed 2-weeks apart was 0·82. We also assessed deviation from a theoretical optimum branching coefficient (14, 23). The optimum branching coefficient for each bifurcation varied between 1·00 and 1·26 according to the asymmetry index, which is the ratio of the smaller daughter diameter over the larger daughter diameter (24).

### Arteriolar branching angles

**Fig 2:**
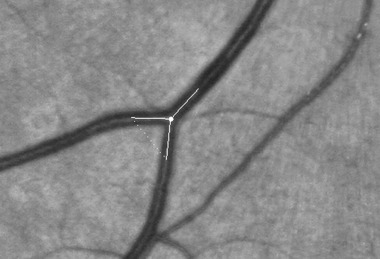
Example of measurement of retinal vessel branching angle. The lines denoting the direction of the branches were produced by the semi-automated software, which tracked down each vessel and the angle subtended by the daughter branches calculated with the cosine rule. Please note that for illustrative purposes the vessel demonstrated in the image is a venule. We performed measurements on retinal arterioles.

A trained grader identified the five most proximal measurable bifurcations to the optic disc and the software tracked down each vessel 2 parent vessel widths from the bifurcation [where turbulent flow becomes laminar after the bifurcation (25)] and drew a line reflecting the course of the vessel. The branching angle was calculated using the cosine rule (Fig. 2). Because of a large variation in the angles within each eye, we only included patients in the analysis in whom we were able to perform five angle measurements to provide a reliable average. As we could not assume a normal distribution of angles within each eye, we took the median of the five angles from each eye. The correlation between angles in the left and right eye was poor (Pearson's correlation coefficient 0·23 for 27 randomly chosen patients); we therefore measured angles in both eyes. We then took the mean of these two values to give an angle measurement for each patient.

We also measured deviation from the optimum branching angle (theoretically calculated as 75°) (14, 23) for each bifurcation and we assessed the median deviation from the optimum for each eye. In a random sample of 10 photographs graded 2-weeks apart, the intrarater class correlation coefficient for the median angle was excellent at 0·961.

### Statistical analysis

All analyses were performed within Minitab (version 14, Minitab Inc., State College, PA, US). We compared the baseline characteristics between the two stroke groups using *t*-tests, Mann–Whitney *U*-tests and differences in proportions. The branching coefficients and branching angles were normally distributed between patients, as were the deviations from the optimum branching coefficient and angles (after square root transformation); we therefore performed multivariable linear regression with branching coefficient and branching angles as the continuous outcomes and vascular risk factors, stroke subtype and WMH as the independent explanatory variables. We set an *α*-level for significance of 0·05.

This study was approved by the Local (Lothian) Research Ethics Committee and all patients provided written informed consent.

## Results

We recruited 205 patients [mean age 68·0-years, standard deviation (SD) 11·6]. There were 104 lacunar strokes (51%) and 101 cortical strokes (49%), and 135 patients were male (66%). We could not measure at least three branching coefficients in 24 patients (due to the poor quality of the photograph, a paucity of bifurcations in the field of view or local anatomical variations precluding computer measurements of vessel widths). Therefore, 181 patients were included in the analysis of branching coefficients. We were not able to measure five branching angles in at least one eye of 61 patients and therefore included 144 patients in the analysis of branching angles. The 24 patients excluded from the branching coefficient analysis were older [74·5 (SD 8·52) years vs. 67·1 (SD 11·7) years] and the 61 patients excluded from the branching angle analysis more often had hypertension (75 vs. 56%) but did not differ in other respects. The baseline characteristics of the 181 patients with at least three branching coefficients are shown in Table 1.

**Table 1:** Baseline characteristics of the patients by ischaemic stroke subtype

Characteristic	Lacunar stroke	Cortical stroke	*P* value for difference
*n*	94	87	
Mean age (SD), years	65·2 (11·5)	69·2 (11·5)	0·02
Male, *n* (%)	53 (56%)	62 (71%)	0·04
AF, *n* (%)	4 (4%)	11 (13%)	0·05
Carotid stenosis >50%, *n* (%)	4 (5%)	10 (12%)	0·08
Median deep WMH Fazekas score (IQR)	1 (1–2)	1 (1–2)	0·97
Median periventricular WMH Fazekas score (IQR)	1 (1–2)	1 (1–1)	0·50
Past medical history of:			
Hypertension, *n* (%)	57 (66%)	53 (56%)	0·21
Diabetes, *n* (%)	18 (19%)	10 (11%)	0·15
Ischaemic heart disease, *n* (%)	13 (14%)	23 (26%)	0·03
Peripheral vascular disease, *n* (%)	3 (3%)	1 (1%)	0·62
Previous stroke/TIA, *n* (%)	20 (21%)	17 (20%)	0·77

AF, atrial fibrillation; WMH, white matter hyperintensity; TIA, transient ischaemic attack; IQR, interquartile range; SD, standard deviation.

### Arteriolar branching coefficients

In the 181 patients, the mean branching coefficient was 1·44 (SD 0·19). There was no difference in the mean branching coefficients between lacunar (1·43, SD 0·17) and cortical stroke (1·44, SD 0·20). On multivariable linear regression (Table 2), both the presence of IHD and an increased periventricular WMH score were significantly and independently associated with increased branching coefficients (representing wider daughters in relation to the parent vessel) and an increased deep WMH score was significantly and independently associated with decreased branching coefficients (representing narrower daughter vessel diameters in relation to the parent vessel). In our prespecified analysis of patients with five branching coefficients measured in an eye (*n*=119), the relationships between branching coefficient and IHD and deep WMH remained but the association between periventricular WMH and branching coefficient was attenuated and became nonsignificant (data not shown). When we looked at deviation from the optimum branching coefficient, we found that the results did not change from those in Table 2.

**Table 2:** Multivariable adjusted associations with absolute retinal arteriolar branching coefficients

Variable	β-Coefficient	*P* value
Lacunar stroke subtype	−0·001	0·96
Age	−0·001	0·70
Deep WMH	−0·076	0·003
Periventricular WMH	0·072	0·006
Past history of:		
Hypertension	−0·020	0·50
Diabetes	−0·032	0·38
Ischaemic heart disease	0·155	<0·001
Stroke/TIA	0·040	0·25
Peripheral vascular disease	0·032	0·73

All values are corrected for the presence of all of the other variables in the table.TIA, transient ischaemic attack; WMH, white matter hyperintensity.

### Arteriolar branching angles

In the 144 patients with five angles measured per eye, we found that the mean branching angle was 84·1°, with an SD of 7·1°. Arteriolar branching angles did not significantly differ between lacunar [mean 85·2° (SD 7·3°)] and cortical stroke [mean 83·0° (SD 7·3°), difference=2·3, 95% CI 0·0 to 4·6, *P*=0·054]. On univariable and also on multivariable analysis, only a history of PVD was associated with increased branching angle (Table 3), but note there were very few patients with PVD. Retinal branching angles were not associated with either deep or periventricular WMH. We assessed deviation from an optimum branching angle of 75° but the associations shown in Table 3 did not change.

**Table 3:** Multivariable adjusted associations with absolute retinal arteriolar branching angles

Variable	β-Coefficient	*P* value
Lacunar stroke subtype	2·22	0·07
Age	−0·03	0·61
Deep WMH score	1·12	0·34
Periventricular WMH score	−0·52	0·65
Past history of:		
Hypertension	1·27	0·33
Diabetes	0·95	0·57
Ischaemic heart disease	0·51	0·75
Stroke/TIA	−0·33	0·83
Peripheral vascular disease	9·05	0·006

All values are corrected for the presence of all of the other variables in the table.TIA, transient ischaemic attack; WMH, white matter hyperintensity.

## Discussion

We have shown that increased retinal arteriolar branching coefficients are associated with increased periventricular WMH and IHD in patients presenting with mild stroke. Decreased retinal arteriolar branching coefficients are associated with increased deep WMH. Branching coefficients are not associated with ischaemic stroke subtype. We have not demonstrated significant associations between retinal arteriolar branching angles and ischaemic stroke subtype, WMH or most other vascular risk factors. No previous studies have assessed retinal vascular geometry within ischaemic stroke subtypes or associations with WMH.

The strengths of this study include prospective recruitment and careful patient assessment at the time of the stroke by an experienced physician with diagnostic MRI graded by an experienced neuroradiologist. Assessment of retinal images was blind to clinical and imaging details. We used a specifically written semi-automated software program to assess retinal vessels to minimise human operator variability, resulting in excellent intrarater repeatability scores. We found that angles did not correlate well between left and right eyes and so measured both eyes where possible. We used patients with cortical stroke as controls to avoid confounding by secondary preventative medications, common vascular risk factors and the presence of stroke, all of which might theoretically affect the appearance of small vessels. Comparison with normal age-matched controls without stroke would not have been appropriate, as then we would only be able to conclude that any differences were due to the presence of risk factors and having any stroke.

We also acknowledge weaknesses. The semi-automated software limited the number of patients that we were able to include because, unlike a human operator, the semi-automated software is not able to make allowances for anatomically difficult vessels, i.e. those with indistinct edges or where a venule is in close proximity to an arteriole. The sample size may not have been large enough to account for interactions between key variables.

It is intriguing that deep and periventricular WMH are associated with opposing directions of altered branching coefficient. A decreased branching coefficient indicates that the daughter vessels are narrower with respect to the parent vessel and an increased branching coefficient indicates that the daughters are wider. Pathological studies have indicated that the mechanism of tissue damage in deep and periventricular WMH may differ (26) as deep lesions may have more ‘ischaemic’ causes while periventricular changes may occur following disruption of the ependymal lining of the ventricles (27). Deep and periventricular WMH may have slightly different associations with vascular risk factors (28) and so, at least for the present, should be considered separately in the assessment of white matter disease (29).

The association between IHD and increased branching coefficients validates previous findings that increased branching coefficients predicted death with IHD. The exact explanation for this is unclear. It is not simply attributable to medication as both our patient groups were taking similar medications and medication is not known to affect retinal vessel widths (30). The true pathophysiological significance of branching coefficients is not known, nor whether these are fixed from birth, alter with age, predispose to or change in the presence of disease. It is therefore difficult to speculate on whether increased branching coefficients might predispose to or be a response to large artery disease. Further studies are needed to examine this finding.

We found no associations with branching angles (the association with PVD is based on few patients), consistent with some previous studies finding no link between angles and hypertension (31), peripheral vascular disease (15) and death with IHD and stroke (16). It is possible that angles may not predispose to or change in response to systemic disease. The poor correlation between the left and the right eye further questions whether angles have anything to do with systemic disease. Previous studies either looked at one eye only or combined eyes (16), thereby assuming that angles do not differ between left and right, or did not specify which eye was measured.

In our cohort, the observed mean of 1·43 (SD 0·19) was higher than the optimum theoretically derived branching coefficient of 1·26. As the majority of our patients had positive deviations from the theoretical optimum, our results did not alter when we assessed deviation from the optimum branching coefficient rather than absolute values, consistent with other studies (16). In study populations where the mean branching coefficient (or branching angle) is closer to the theoretical optimum, assessing deviation leads to more diverse results (14). However, not all theories attempting to explain biological systems hold true *in vivo*. Data are too sparse to know whether optimality of branching coefficients and angles differ between arteriolar beds or patient populations. Further studies should assess both absolute and deviation from optimum values focusing on different vascular beds, in response to pharmacological challenges, at different ages and in the presence of different diseases to assess the real implications of vascular geometry.
